# Application of Pulsed Field Gel Electrophoresis for Study of Genetic Diversity in *Mycobacterium tuberculosis* Strains Isolated From Tuberculosis Patients

**DOI:** 10.5812/jjm.9963

**Published:** 2014-05-01

**Authors:** Azar Dokht Khosravi, Shideh Vatani, Mohammad Mehdi Feizabadi, Effat Abasi Montazeri, Abbas Jolodar

**Affiliations:** 1 Department of Microbiology, School of Medicine, Ahvaz Jundishapur University of Medical Sciences, Ahvaz, IR Iran; 2 Health Research Center, Infectious and Tropical Diseases Research Center, Ahvaz Jundishapur University of Medical Sciences, Ahvaz, IR Iran; 3 Department of Microbiology, School of Medicine, Tehran University of Medical Sciences, Tehran, IR Iran; 4 School of Veterinary Medicine, Shahid Chamran University, Ahvaz, IR Iran

**Keywords:** *Mycobacterium tuberculosis*, Tuberculosis, Genotyping Techniques

## Abstract

**Background:**

*Mycobacterium tuberculosis* genotyping can effectively improve tuberculosis (TB) control programs by controlling disease transmission. Pulsed field gel electrophoresis (PFGE) is a particularly powerful tool for determination of clonal identity of bacteria providing information for understanding and controlling the spread of disease.

**Objectives:**

The aim of present study was to investigate the genetic diversity of *M. tuberculosis* strains in Khuzestan province by the PFGE technique.

**Patients and Methods:**

In total, 80 *M. tuberculosis* positive cultures were obtained from tuberculosis patients. PFGE was performed on 60 PCR-confirmed isolates by using *DraI* and *XbaI* restriction enzymes according to standard protocols. Plugs containing digested DNA were then loaded on agarose gels and run using contour-clamped homogenous electric fields.

**Results:**

Fifty distinct DNA banding patterns were obtained by digestion of DNA with *DraI* and 38 DNA banding patterns by digestion with *XbaI* restriction enzymes. The patterns comprised of 17 different clusters in which cluster I was the major one, containing six strains. Three clusters contained three strains each and the 13 remaining clusters comprised of two strains each. Digestion with *DraI* yielded 15-20 DNA fragments with 50-485 kb size, while digestion by *XbaI* produced DNA fragments with a size smaller than 50-242 kb.

**Conclusions:**

Despite the ability of PFGE for study of genetic diversity of many mycobacterial species and it being considered as a robust and useful tool, in this study we only found a 15% epidemiological relationship amongst the isolates. Thus, for higher discrimination of genotypic clusters among *M. tuberculosis* clinical isolates, the application of more sophisticated complementary techniques is required.

## 1. Background

Tuberculosis remains a major health problem worldwide. An estimated 1700 million people, one-third of the world's population, are infected or have been infected with *Mycobacterium tuberculosis*, with an estimated eight million new cases and two million deaths occurring every year (1). Molecular epidemiology, the study of distribution and determinants of disease occurrence in human populations using molecular techniques, is a blend of molecular biology and epidemiology. Tracking a particular strain of *M. tuberculosis,* as it moves through a susceptible population, has been almost impossible, since in most cases there are no strain distinguishing characteristics (2, 3). 

Rarely do antibiotic susceptibility patterns, serotyping, biotyping, and bacteriophage typing allow strain identification (4). With advances in molecular biology, techniques for strain specific epidemiologic studies of tuberculosis are becoming available, such as the DNA fingerprinting technique which uses radioactive probes of the insertion sequence *IS6110* to detect differences in genomic DNA digests of *M. tuberculosis* strains (5). Molecular typing is now widely used to aid and supplement conventional epidemiological studies of mycobacterial diseases. 

Pulsed field gel electrophoresis (PFGE), by which the entire genome can be represented as a distinct pattern of DNA restriction fragments, is a particularly powerful tool in epidemiology for the determination of clonal identity of bacteria providing information for understanding and controlling the spread of disease (6). However, application of PFGE to study mycobacterial diseases has been limited because isolation of high-quality genomic DNA from mycobacterial sources has proved problematic. The mycobacterial cell wall/envelope is highly specialized with a wide diversity of lipids which shield it from environmental stresses and also make it impermeable to many chemicals (7). In addition, mycobacterial cells are prone to aggregation forming clumps. Thus, standard protocols for preparation of DNA for PFGE do not work with any degree of efficiency (6). 

Variability in the genomic copy number of the repetitive sequence *IS6110* and polymorphism of the flanking *PvuII* restriction site has been exploited to generate strain-specific genotypes for *M. tuberculosis*. The *IS6110* genotyping system has been widely used in epidemiologic studies of tuberculosis. However, the inadequacies and complexity of restriction fragment length polymorphism (RFLP) analysis with *IS6110* have created the need for secondary typing techniques. Isolates of *M. tuberculosis* containing a few copies of *IS6110* present a problem, as limited information is available to infer genetic relatedness (8).

PFGE has been widely used to type various microorganisms in both outbreak and population-based studies and is available in many clinical laboratories (9, 10). Although, to date, PFGE has not been commonly employed in epidemiological investigations of *M. tuberculosis*. Most published PFGE protocols for *M. tuberculosis* are technically challenging (8).

## 2. Objectives

The aim of present study was to investigate the genetic diversity of *M. tuberculosis* strains in Khuzestan province of Iran, by PFGE to reevaluate the discriminatory power of the technique.

## 3. Patients and Methods

### 3.1. Clinical Isolates

In total, 80 *M. tuberculosis* positive cultures were obtained from 100 patients referred to the tuberculosis reference laboratory of Khuzestan Province, Iran, from October 2008 to July 2010. These isolates, were originally recovered from patients' specimens on Lowenstein Jensen (LJ) medium slots (Pasteur Institute, IR Iran), after decontamination of sputum samples and incubation at 37°C for 4-6 weeks. For DNA extraction, a few colonies from the surface of LJ medium were harvested and suspended in 500 μL 10 mM Tris-HCl/1 mM EDTA [pH = 7] and inactivated at 100°C for 30 minutes (11). After centrifugation at 12000 rpm for 15 minutes, the supernatant was used for PCR. Standard *M. tuberculosis* strain (H37Rv) was obtained from the Pasteur Institute, Iran, and prepared according to the protocol for clinical isolates.

### 3.2. PCR Technique

Identification of the *M. tuberculosis* complex (MTBC) was performed with PCR amplification of the *IS6110* element, specific for the MTBC, using the forward primer (5΄CCTGCGAGCGTAGGCGTCGG3΄) and reverse primer (5΄CTCGTCCAGCGC CGCTTCGG3΄) which amplify a 123 bp fragment (12, 13).

### 3.3. PFGE

#### 3.3.1. Preparation of Organisms for DNA Extraction

This was done according to a recent previously reported extraction method (14). In brief, after the preparation of bacterial suspension and heat killing at 80**°**C for 30 minutes, the pre-treated bacterial sediment was suspended in EC buffer (pH = 7.6). For standardizing the suspension, the optical density (McFarland Units) was allowed to reach at least 1 µg/mL (OD 550 nm 0.25) and not greater than 4 (OD 550 nm 1.00) as determined using a densimat (suitable OD was 2/99 at 600 nm in this study). All steps for growth and harvesting of bacteria were carried out inside a class II biological safety cabinet.

#### 3.3.2. Plug Preparation

The suspension was boiled in a water bath for 15 minutes. To each sample, 20 µL of EC buffer containing 0.6 mg of lysozyme (30 mg per 1 mL) was added. After incubation of the cell suspension in a 54°C water bath for 5-10 minutes, it was mixed with an equal volume of 2% low-melting-point agarose (LMP), and the mixture was poured into plug molds. The plugs were kept for ten minutes at room temperature and then for 20 minutes at 4°C to solidify. Plugs were then transferred to 50 mL falcon tubes containing 10 mL EDTA 0.5 M and Sodium Lauryl Sarkosyl 10% and placed in a 37°C water bath overnight with gentle shaking. The plugs were then transferred to 10 ml ESP buffer (lysis buffer II) containing 0.5 mM EDTA (pH = 8.5-9), 1% Sarkosyl plus 150 µL proteinase K (20 mg/mL) and incubated in a water bath at 50°C overnight with gentle shaking. After five times of washing with 10 mL of TE buffer, the plugs were ready for further processing. All the reagents for the PCR reaction and PFGE were purchased from the Cinnagen Company, Iran.

#### 3.3.3. Restriction Endonuclease Digestion

Agarose plugs were cut with a sterile scalpel to fit the size of the combs of the gel casting (3-4 mm). They were washed in restriction buffer at 4°C for 30 minutes. The restriction enzymes (REs) used for PFGE sample preparation included *DraI* and *XbaI* (Fermentas, Canada) with the appropriate restriction buffer. The restriction digestion steps were done separately by adding 30 U of *XbaI* enzyme followed by incubation at 37°C for four hours and *DraI* enzyme followed by an overnight incubation at 37°C.

#### 3.3.4. PFGE Technique

Plugs containing digested DNA were loaded in wells of 1% agarose gel (Cinnagen Company, Iran) and run in 0.5X TBE buffer. PFGE was carried out with contour-clamped homogeneous electric fields (CHEF DRIII, Bio-Rad, USA) at 14°C for 24 hours at 180 v and 20 A. The electrophoretic conditions for *DraI* were as follows; the pulse time was ramped from 5 to 15 seconds for 16 hours and then from 60 to 70 seconds for 8 hours; and for *XbaI* the pulse time was ramped from 5 to 15 seconds for 16 hours and then from 1 to 20 seconds for 8 hours. The gel was stained with 0.5 mg/mL ethidium bromide for 20 minutes and de-stained in water for 20 minutes. Bacteriophage lambda chromosomal DNA was used as a molecular weight marker (MBI, Fermentas Germany). Computer assisted analysis was performed for data interpretation (SPSS version 17).

## 4. Results

**Figure 1.**
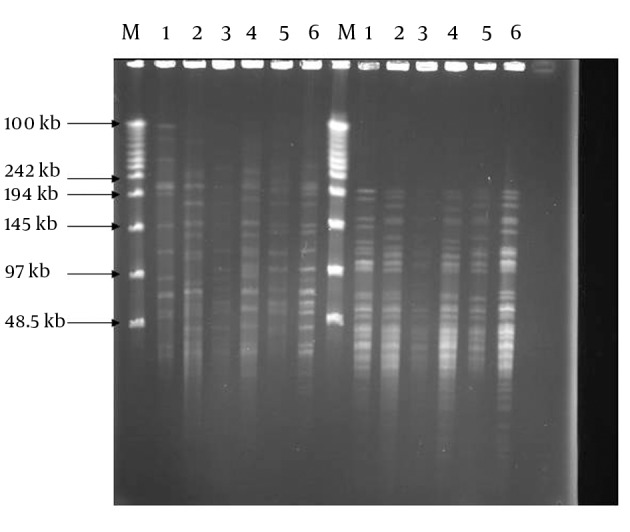
PFGE Genomic Patterns After Digestion With DraI and XbaI Restriction Enzymes M. DNA weight marker, 1. BCG; 2. *M. tuberculosis* H37Rv; 3-6 clinical isolates: *DraI* digests (left); *XbaI* digests (right)

Positive cultures were obtained from 80 patients, of which 44 were male and 36 were female with mean age of 40.6. Sixty out of 80 samples were positive by the PCR method, thus they belonged to the *M. tuberculosis* complex. These isolates were subjected to PFGE in the next step. By using PFGE, 50 distinct DNA banding patterns were obtained by digestion of DNA with *DraI* and 38 DNA banding patterns by digestion with *XbaI* REs (Figure 1). The patterns comprised of 17 different clusters, where cluster one was the major, containing six strains. Three clusters contained 3 strains each and the 13 remaining clusters comprised of two strains each (Table 1). Digestion with *DraI* yielded 15-20 DNA fragments with size of 50-485 kb, while digestion with *XbaI* produced DNA fragments smaller than 50-242 kb. The DNA banding patterns were able to distinguish some of the epidemiological criteria of tested MTB strains.

**Table 1.** PFGE Clusters of *M. tuberculosis* Isolates Characterized by DraI Digestion ^a^

Clusters	Strains	Epidemiological Relationship
**I**	6 (12)	5 (83.3)
**II**	3 (6)	-
**III**	3 (6)	2 (66.6)
**IV**	3 (6)	-
**V**	2 (4)	2 (66.6)
**VI to XVII**	24 (48)	-
**Non cluster**	19	-

^a^ Data are presented as No. (%).

## 5. Discussion

For large scale epidemiological studies the portability and standardization of *IS6110* restriction fragment length polymorphism (RFLP) means that it remains the gold standard technique (15). PFGE was initially designed to simplify RFLP. The method uses a less frequent cutting enzyme that generates high molecular weight fragments and allows separation of these fragments under special conditions in PFGE (5).

In this study, by using an improved technique of DNA extraction, we found that considerable diversity is revealed by PFGE typing using each of the two restriction enzymes (RE). Each *M. tuberculosis* isolate gave a readily large restriction fragment pattern when its chromosomal DNA was digested with *DraI* or *XbaI*, and subjected to PFGE. The location of RE *DraI* (TTTAAA) is within the *IS6110* sequence. Since these recombinant sequences are transmissible, the location and copy number is quite variable in the genome of different strains. It is likely that diversity in patterns after digestion with *DraI* is due to variety in number and copies of *IS6110* (5).

*DraI* produced larger size fragments and the widest molecular weight range. In contrast to previous investigators’ results (16) by which low genetic diversity among *M. tuberculosis* isolates was reported, the degrees of diversity revealed by these enzymes were high in the present study, which is in agreement with the findings of Feizabadi et al. (9). Besides, in this study, similar to that reported by Zhang et al. (5), the isolates in some clusters showed similar patterns, while the isolates in different clusters revealed various patterns. 

Cluster I comprised of strains majorly (five of six) isolated from patients of identical living locations and previously proved by different techniques to be epidemiologically related (17). Two of the strains in cluster III and two strains in cluster IV were also epidemiologically related. However we could not find any relationship between other strain members of the other clusters (Table 1). The 19 remaining isolates were not classified in to any of the characterized clusters. The patterns produced by *XbaI* digestion had more discriminatory capacity by yielding more fragments. However, they sometimes overlapped and made the analysis difficult. Feizabadi et al. (9) concluded that this enzyme is mainly used for confirmation of the results of *DraI* digestion. We previously reported the genotyping of *M. tuberculosis* isolates in the same area by using the MIRU-VNTR technique (17), though we found the earlier method was more discriminatory for typing of the isolates. 

The main limitation of this technique is that the small polymorphism characteristics for different strains will not always produce sufficient discrimination (18). However, this technique has proved to be a useful tool with high discriminatory power for typing of non-tuberculous mycobacteria in the recent years (19, 20). In the present study by using the applied technique, we were unable to discriminate all the *M. tuberculosis* strains among the clinical isolates and we only found nine isolates with an epidemiological relationship amongst all examined isolates (15%). Previously published reports have presented conflicting views on the ability of PFGE to demonstrate genetic diversity and some studies demonstrated the different discriminatory powers of various REs (5, 9).

As mentioned earlier, nowadays, the studied technique is mainly used for genotyping of non-tuberculous mycobacteria, and our literature review did not generate much updated work outside and inside Iran similar to the present study. In summary, despite the ability of PFGE for the study of genetic diversity of many mycobacterial species and it being considered as a robust and useful tool, yet, for higher discrimination of genotypic clusters among *M. tuberculosis* clinical isolates, the application of more sophisticated complementary techniques are required.
